# Effect of Mn^2+^/Zn^2+^/Fe^3+^ Oxy(Hydroxide) Nanoparticles Doping onto Mg-Al-LDH on the Phosphate Removal Capacity from Simulated Wastewater

**DOI:** 10.3390/nano12203680

**Published:** 2022-10-20

**Authors:** Diana Guaya, Hernán Cobos, César Valderrama, José Luis Cortina

**Affiliations:** 1 Department of Chemistry, Universidad Técnica Particular de Loja, Loja 110107, Ecuador; 2 Department of Chemical Engineering, BarcelonaTECH-UPC, 08019 Barcelona, Spain; 3 Barcelona Research Center for Multiscale Science and Engineering, 08930 Barcelona, Spain

**Keywords:** Mg-Al-LDH, Mn^2+^/Zn^2+^/Fe^3+^, composite, phosphate, equilibrium, kinetic, thermodynamics

## Abstract

A parent Mg-Al-LDH was upgraded in its adsorption properties due to the incorporation of tri-metal species oxy(hydroxide) nanoparticles obtaining Mn^2+^/Zn^2+^/Fe^3+^/Mg-Al-LDH composite for the phosphate recovery from simulated urban treated wastewater. The physicochemical properties of the synthesized Mn^2+^/Zn^2+^/Fe^3+^/Mg-Al-LDH make promising for real application without being environmentally harmful. The performance of Mn^2+^/Zn^2+^/Fe^3+^/Mg-Al-LDH composite was evaluated through batch adsorption assays. The support of iron, manganese, and zinc (oxy)hydroxide nanoparticles onto the parent Mg-Al-LDH structure was performed by precipitation, isomorphic substitution, and complexation reactions. The main improvement of the Mn^2+^/Zn^2+^/Fe^3+^/Mg-Al-LDH composite was the highest phosphate adsorption capacity (82.3 mg∙g^−1^) in comparison to the parent Mg-Al-LDH (65.3 mg∙g^−1^), in a broad range of concentrations and the effective phosphate adsorption at neutral pH (7.5) near to the real wastewater effluents conditions in comparison to the conventional limitations of other adsorbents. The effectiveness of Mn^2+^/Zn^2+^/Fe^3+^/Mg-Al-LDH composite was higher than the conventional metal LDHs materials synthesized in a single co-precipitation step. The phosphate adsorption onto Mn^2+^/Zn^2+^/Fe^3+^/Mg-Al-LDH composite was described to be governed by both physical and chemical interactions. The support of Mn^2+^/Zn^2+^/Fe^3+^ oxy(hydroxide) nanoparticles over the parent Mg-Al-LDH was a determinant for the improvement of the phosphate adsorption that was governed by complexation, hydrogen bonding, precipitation, and anion exchange. The intra-particular diffusion also described well the phosphate adsorption onto the Mn^2+^/Zn^2+^/Fe^3+^/Mg-Al-LDH composite. Three specific stages of adsorption were determined during the phosphate immobilization with an initial fast rate, followed by the diffusion through the internal pores and the final equilibrium stage, reaching 80% of removal and the equilibrium within 1 h. The Mn^2+^/Zn^2+^/Fe^3+^/Mg-Al-LDH was strongly selective towards phosphate adsorption in presence of competing ions reducing the adsorption capacity at 20%. The Mn^2+^/Zn^2+^/Fe^3+^/Mg-Al-LDH has limited reusability, only 51% of the adsorbed phosphate could be recovered in the second cycle of the adsorption-desorption process. Around 14% of phosphate was loosely-bond to Mn^2+^/Zn^2+^/Fe^3+^/Mg-Al-LDH which brings the opportunity to be a new source of phosphorus. The use of eluted concentrates and the final disposal of the exhausted adsorbent for soil amendment applications can be an integral nutrient system (P, Mn, Zn, Fe) for agriculture purposes.

## 1. Introduction

Water is an essential natural resource, however, there are fewer safe freshwater sources worldwide. The increase of urbanization is traduced on more water consumers which is the cause of deterioration of water quality since wastewater discharges contain excessive amounts of nutrients (e.g., phosphates, nitrates, and ammonia) [1]. Phosphate is the ionic form of phosphorous in water; naturally, the phosphate enters the water bodies from weathering of rocks and the leaching of soil and rain [2]. Also, phosphate comes from agricultural runoff and domestic sewage (e.g., detergents and household wastes). The anthropogenic phosphorous input is responsible for the acceleration of eutrophication [3]; which is the enrichment process of nutrients in any aquatic body that outcomes in the incontrollable growth of aquatic plants. The algae’s death and decay result in the reduction of dissolved oxygen [1]. Then, eutrophication is a global problem in all aquatic environments.

One target of sustainable development proposed by the United Nations is the improvement of water quality by reducing pollution. However, many countries do not even have any sewage treatment systems or even it is deficient [4]. The use of wastewater seems to be the most important source for nutrient recycling since common wastewater treatment systems have a limited nutrient removal efficiency. Conventionally, phosphate removal includes biological, chemical, and physical treatment processes; however, they are expensive and non-effective in removing traces [5]. The adsorption treatment for wastewater has attracted scientists’ attention as one of the most promising strategies for wastewater purification. Several organic and inorganic harmful pollutants have been efficiently removed from water and wastewater using adsorption techniques [6,7]. Thus, the adsorption process is considered optimal for phosphate removal [8] due to its high efficiency, sensitivity, and selectivity. Also, the easy operation and maintenance of the adsorption systems have been reported as the most important advantages.

Several, organic and inorganic adsorbents have been largely used for phosphate removal, such as: industrial [9] and agricultural wastes [10], polymeric exchangers, natural zeolites, natural clays, and also other synthetic material (e.g., zeolites, hydrotalcites) [2,11]. However, nowadays research efforts are focused on the development of high-quality adsorbents with high performance, that allow regeneration and quick final disposal. The advent of nanotechnology has become strategic to synthesize nanoparticles and composites with high-performance properties. Unfortunately, within the manufacture of several nano-adsorbents, some harmful chemicals are necessarily becoming an environmental problem [12]. Therefore, environmentally friendly adsorbents are also desirable for real application at full scale. Within this background, the LDHs materials have been reported to be non-toxic by in vitro essays since they were used for drug delivery purposes [13]. Thus, we considered hydrotalcites (LDHs) as an ideal candidate for adsorbent for phosphate removal since maximum adsorption capacities are up to three times that reported for natural clays and zeolites [2,14].

The hydrotalcites are layered double hydroxides (LDHs) which belong to the minerals of the anionic clay family. The LDHs are hydrated minerals that can be easily synthesized as hydrotalcite-like compounds by rarely found in nature [15]. The LDHs consist of a positively charged brucite-like octahedral layer that is formed by partial substitution of a trivalent metal for a divalent one [16]. LDHs can be represented by the general formula [M^2+^_1−x_ M^3+^_x_ (OH)_2_]^x+^(A^n^^−^)_x/n_·mH_2_O. M^2+^ represents the divalent cations (e.g., Mg^2+^, Zn^2+^, Ni^2+^, Fe^2+^, Cu^2+^), M^3+^ denotes the trivalent cations (e.g., Al^3+^, Fe^3+^, Cr^3+^) and A^n^^−^ is the anion (e.g., CO_3_^2^^−^, NO^3^^−^, Cl^−^, SO_4_^2^^−^). The value of x is equal to the molar ratio of M^3+^/(M^2+^ + M^3+^), whereas A is the interlayer anion of valence n [17]. The specific elements occupying the M^2+^, M^3+^, and A^n−^ position of LDHs determine their physicochemical properties. Also, LDHs contain anions and water molecules in the interlayer with high anion exchange capacity. The interlayer anions are exchangeable for other anions with higher selectivity [18]. LDHs provide higher characteristics in comparison to other adsorbents due to their structural stability and crystallinity. LDHs also provide binding sites for pollutant removal at both the external surface and internal surface of each individual hydroxide sheet [19].

Conventionally, the parent Mg-Al-LDHs have been reported as excellent adsorbents for phosphate removal [20,21], but the incorporation of transition metals has been used as an alternative for phosphate removal (e.g., Zn-Al, Fe-Mg) [22,23]. The binary metal combination on LDHs revealed good physicochemical properties for application in water treatment. Conversely, the ternary metal combination LDHs have been reported to provide higher surface area than bimetal ones because LDHs develop a synergistic effect between two divalent metals making it interesting for its adsorption properties [9] of ionic species from water. Thus, the replacement of divalent cations (e.g., Mg^2+^) by trivalent cations (e.g., Al^3+^, Fe^3+^) in the layers of LDHs materials provides a positive charge that improves the phosphate adsorption capacity. Some ternary LDHs have demonstrated effectiveness towards anionic pollutants, such as the arsenate adsorption onto Cu-Mg-Fe-LDH [24], an Mg-Ca-Fe LDH for fluoride removal [25], and a Fe-Mg-Mn-LDH for phosphate removal [26]. Usually, the metallic elements are incorporated during the synthesis of the LDHs materials by the co-precipitation method [19,26]. However, to the best of our knowledge, the incorporation of metals onto LDHs has not been performed over a synthesized Mg-Al-LDH. Thus, we have developed a novel composite incorporating Mn^2+^/Zn^2+^/Fe^3+^ (oxy)hydroxide nanoparticles onto Mg-Al-LDH as a promising adsorbent with high affinity to phosphate. We have considered the doping method could determine the physicochemical properties and hence the efficiency. Also, the combination of the Fe-Mn-Al elements has been proposed considering that published research based on this strategic nutrient system has not been easily found. Thus, the nutrient recovery, which is the purpose of this work, using a Mn^2+^/Zn^2+^/Fe^3+^/Mg-Al-LDH composite was conceived as non-toxic and non-harmful material with the potential for being used as fertilizer. Also, non-harmful elements (e.g., heavy metals) intervene during the synthesis of Mn^2+^/Zn^2+^/Fe^3+^/Mg-Al-LDH composite. This inorganic material after the phosphate adsorption can potentially provide macro and micronutrients in the scenery of final disposal for soil amendment application. The improvement of the physical and chemical properties of soils can be performed without the risk of releasing any harmful pollutants into the water or soil.

In this work, a novel Mn^2+^/Zn^2+^/Fe^3+^/Mg-Al-LDH composite was prepared for phosphate removal from simulated wastewater. We developed a phosphate adsorbent that does not require pH adjustment during treatment, considering the expected value of treated wastewater was in the range of pH values between 6 and 8. Most studies regarding LDHs materials are focused on the phosphate removal mechanism, with little attention given to their possible regeneration. The lack of information about the lifespan and alternatives for the environmentally friendly final disposal was our motivation to perform a complete evaluation of the phosphate adsorption onto Mn^2+^/Zn^2+^/Fe^3+^/Mg-Al-LDH composite. In this study we: (i) obtain a novel Mn^2+^/Zn^2+^/Fe^3+^/Mg-Al-LDH composite for the phosphate removal at a neutral pH range, (ii) estimate the effect of pH on phosphate adsorption, (iii) determine the phosphate maximum adsorption capacity and thermodynamic behavior, (iv) assess the kinetic of phosphate adsorption, (v) investigate the selectivity towards phosphate over competing ions, (vi) evaluate the regeneration and lifespan of Mn^2+^/Zn^2+^/Fe^3+^/Mg-Al-LDH composite in continuous adsorption-desorption cycles and (vii) propose an alternative for final disposal of the exhausted Mn^2+^/Zn^2+^/Fe^3+^/Mg-Al-LDH composite by means of the phosphate fractioning essay verifying the phosphate availability for plants in case of a soil amendment application. We considered that the overall information here detailed is important for further real implementation in pilot plants or large-scale systems.

## 2. Materials and Methods

### 2.1. Materials

The reagents used in this study included: Mg(NO_3_)_2_·6H_2_O (Loba Chemie Pvt. Ltd., Tarapur, Maharashtra, India), Al(NO_3_)_3_·9H_2_O (Fisher Scientific, Waltham, MA, USA), HCl (Fisher Scientific, Waltham, MA, USA), NaOH (EMSURE®, Merck KGaA, Darmstadt, Germany), NaH_2_PO_4_·2H_2_O (Loba Chemie Pvt. Ltd., Tarapur, Maharashtra, India), MnCl_2_·4H_2_O (Loba Chemie Pvt. Ltd., Tarapur, Maharashtra, India), FeCl_3_·6H_2_O (Loba Chemie Pvt. Ltd., Tarapur, Maharashtra, India), and ZnCl_2_ (Loba Chemie Pvt. Ltd., Tarapur, Maharashtra, India). All the chemicals used in the test were analytically pure.

### 2.2. Synthesis of Mn^2+^/Zn^2+^/Fe^3+^/Mg-Al-LDH Composite Adsorbent

The parent Mg-Al-LDH was synthesized by an adaptation of the conventional co-precipitation method [20]. In 200 mL of oxygen-free deionized water solution it was dissolved Mg(NO_3_)_2_·6H_2_O (0.42 M) and Al(NO_3_)_3_·9H_2_O (0.21 M) using a [Mg^2+^]/[Al^3+^] = 2 ratio. The solution was added dropwise into a flask and simultaneously, a solution of NaOH (2 M) was added under vigorous magnetic stirring to maintain a constant pH of 9.5. The suspension was transferred to an ultrasound generator for 30 min. The suspension was aged for 24 h at room temperature. Then, the supernatant was discarded and solids were washed and vacuum filtered until the effluent solution was at pH 7. Finally, the resultant parent Mg-Al-LDH solid was dried at 80 °C for 14 h.

To prevent the oxidation of metal ions, oxygen-free deionized water was used to prepare the modification solution of parent Mg-Al-LDH. A weighted amount of 50 g of Mg-Al parent hydrotalcite was modified in a combination of 250 mL of oxygen-free deionized water red dark solution containing: MnCl_2_·4H_2_O (0.1 M), FeCl_3_·6H_2_O (0.1 M) and ZnCl_2_ (0.1 M). It was used as an adaptation of the co-precipitation method (oxy)hydroxide nanoparticles [27] for the incorporation of Mn^2+^/Zn^2+^/Fe^3+^ onto the previously synthesized Mg-Al-LDH. The pH value of the suspension was continuously adjusted to pH 9 while the temperature was maintained at 90 °C with a stirring speed of 100 rpm for 5 h. The solid suspension was washed, and vacuum filtered until the supernatant showed no coloration and pH 7 was obtained. Finally, the solids of Mn^2+^/Zn^2+^/Fe^3+^/Mg-Al-LDH composite were dried at 80 °C for 14 h and stored for further experimentation.

### 2.3. Physicochemical Characterization of Materials

A powder X-ray diffractometer was used for the characterization of the parent and modified hydrotalcite samples. The X-ray diffraction (XRD) patterns were acquired on a powder X-ray Diffractometer (D8 Advance A25 Bruker, Karlsruhe, Germany) with a Cu Kα anode (λ = 0.1542 nm) operating at 40 kV and 40 mA. The chemical composition and morphology of the samples were determined using a field emission scanning electron microscope (JEOL, Peabody, MA, USA JSM-7001F, Peabody, MA, USA) coupled to an energy-dispersive spectroscopy system (Oxford Instruments X-Max, Oxford, UK, Resolution 129 eV). The SEM-EDX analysis was replicated three times and the average data is reported using the standard deviation. The nitrogen adsorption method was used to determine the specific surface area of the adsorbents with an automatic adsorption analyzer (Micrometrics Chemisorb 2720, Norcross, GA, USA) using the single-point nitrogen gas adsorption technique. The infrared absorption spectra were recorded in the range of 4000–550 cm^−1^ with a Fourier Transform FTIR spectrometer (4100 Jasco, Easton, MD, USA). The point of zero charge (PZC) by the pH drift method of the hydrotalcite samples in the modified form was determined. The Mn^2+^/Zn^2+^/Fe^3+^/Mg-Al-LDH composite was equilibrated at different ionic strength solutions in the range of pH 3–10, as it was described in previous work [3]. The assay was replicated three times for each sample and the average data is reported.

### 2.4. Batch Adsorption Studies

The adsorption tests were performed using a synthetic phosphate solution by preparing a NaH_2_PO_4_∙2H_2_O stock solution (1000 mg∙L^−1^) in deionized water. The pH of the phosphate solution used for each essay was adjusted by the addition of 0.1 M HCl or 0.1 M NaOH. In each essay, it was determined the initial and the final phosphate concentration. It used the standard methods for the examination of water and wastewater, specifically the vanadomolybdophosphoric acid colorimetric method (4500-P C) [28]. It also used a Thermo Scientific Ionic Chromatograph (Dionex ICS-1100 and ICS-1000, Thermo Fisher Scientific, Waltham, MA, USA) for anion and cation determination.

#### 2.4.1. Effect of pH on Phosphate Adsorption

A measured amount of 0.05 g Mn^2+^/Zn^2+^/Fe^3+^/Mg-Al-LDH composite sample was equilibrated in 25 mL of phosphate solutions containing (25 mg∙L^−1^∙PO_4_^3−^) at pH values from 4 to 11. Centrifuge tubes in a rotatory stirrer, at 100 rpm at room temperature ~20 °C, were used. Afterward, the suspension was centrifuged for 10 min and the supernatant was filtered through 0.45 μm cellulose membrane before analysis of the liquid phase. The adsorption capacity of each essay was calculated by using Equation (1)

(1) $$q_{e}=\frac{v\times \left(c_{o}-c_{e}\right)}{w}$$

where $q_{e}$ was the equilibrium adsorption capacity (mg∙g^−1^∙PO_4_^3−^), v was the volume of phosphate solution (L), $c_{o}$ and $c_{e}$ were the initial and equilibrium concentration of the phosphate solution (mg∙L^−1^∙PO_4_^3−^), and w was the mass of the Mn^2+^/Zn^2+^/Fe^3+^/Mg-Al-LDH (g). The essays were performed in triplicate and the average values were reported.

#### 2.4.2. Maximum Phosphate Adsorption Capacity

A measured amount (0.05 g) of Mn^2+^/Zn^2+^/Fe^3+^/Mg-Al-LDH composite sample was equilibrated in 25 mL of phosphate solutions containing (10, 25, 50, 100, 250, 500, and 1000 mg∙L^−1^∙PO_4_^3^^−^ at pH 7.5 which is the expected conditions of real treated wastewater). The essays were performed by triplicate in centrifuge tubes in a rotatory stirrer at 100 rpm at 20 °C, 25 °C, and 30 °C. The resultant suspension was centrifuged for 10 min and the supernatant was filtered through 0.45 μm cellulose membrane before analysis of the liquid phase. The experimental equilibrium phosphate adsorption data were fitted to the two isotherm models. The Langmuir and Freundlich [29] isotherm models are conventionally used to describe the macroscopic adsorption data. The Langmuir model describes the homogeneous sorption sites with equal affinity, accordingly, while the Freundlich isotherm model explains adsorption onto heterogeneous sorption sites [30]. The Langmuir model is represented in the linearised form by Equation (2).

(2) $$\frac{c_{e}}{q_{e}}=\frac{c_{e}}{q_{m}}+\frac{1}{k_{L}q_{m}}$$

where $q_{m}$ is the maximum adsorption capacity (mg∙g^−1^∙PO_4_^3−^), $k_{L}$ Langmuir adsorption constant (L∙mg^−1^). In the Langmuir isotherm model, the favourability of the adsorption process is defined by the separation factor $r_{L}$ when 0 < $r_{L}$< 1 and can be calculated by Equation (3). It is a dimensionless constant that explains the Langmuir isotherm shape.

(3) $$r_{L}=\frac{1}{1+k_{L}c_{0}}$$

The Freundlich model is represented in the linearised form by Equation (4).

(4) $$\ln q_{e}=\ln\;k_{F}+\frac{1}{n}\ln c_{e}$$

where $k_{F}$ (mg∙g^−1^) is the maximum adsorption capacity (mg∙g^−1^∙PO_4_^3−^) and n were the Freundlich constant.

#### 2.4.3. Adsorption Thermodynamics

The thermodynamic studies allowed the prediction of adsorption mechanisms by chemical and physical interactions. The experimental data were fitted according to the parameters of the thermodynamic laws described by Gibbs free energy (∆G^0^, kJ∙mol^−1^), enthalpy (∆H^0^, kJ∙mol^−1^), and entropy (∆S^0^, kJ∙mol^−1^∙K^−1^) conventionally used, determined from Equations (5) and (6) [31].

(5) $$∆G^{0}=-RT\ln k_{c}$$

The relationship between ∆G^0^, ∆H^0^, and ∆S^0^, is obtained as Equation (6), the well-known van ’t Hoff equation.

(6) $$\ln k_{c}=\frac{-∆H^{0}}{R}\times \frac{1}{T}+\frac{∆S^{0}}{R}$$

where $k_{L}$ (L∙mg^−1^) is the Langmuir constant and which could be obtained as a dimensionless parameter. The $k_{c}$ is obtained as a dimensionless parameter by multiplying $k_{L}$ by a molecular weight of adsorbate ($M_{w}$, g∙mol^−1^) and then by factors 1000 and 55.5 which is the number of moles of pure water per liter, described in Equation (7) [32]. The R is the universal gas constant (8.314 J∙mol^−1^∙K^−1^), and T is the absolute temperature (K).

(7) $$k_{c}=k_{L}\times M_{w}\times 1000\times 55.5$$

#### 2.4.4. Kinetic Behavior of Phosphate Adsorption

A measured amount of 0.05 g Mn^2+^/Zn^2+^/Fe^3+^/Mg-Al-LDH composite sample was equilibrated in 25 mL of phosphate solutions containing (100 mg∙L^−1^∙PO_4_^3^^−^ at pH 7.5 which is the expected conditions of treated wastewater). The essays were performed by triplicate in centrifuge tubes in a rotatory stirrer at 100 rpm at room temperature ~20 °C. The centrifuge tubes were withdrawn at given times (e.g., 0.5, 1, 2.5, 10, 30 s until 24 h) and quickly separate from the Mn^2+^/Zn^2+^/Fe^3+^/Mg-Al-LDH composite sample by filtration. The resultant suspension was centrifuged for 10 min and filtered through 0.45 μm cellulose membrane before the determination of phosphate content in the liquid phase. The phosphate adsorption capacity at time t was calculated using Equation (8).

(8) $$q_{t}=\frac{v\times \left(c_{o}-c_{t}\right)}{w}$$

where $q_{t}$ was the adsorption capacity as a function of time (mg∙g^−1^∙PO_4_^3−^) and $c_{t}$ was the concentration of the phosphate solution at a specific time (mg∙L^−1^∙PO_4_^3−^). The phosphate removal capacity of Mn^2+^/Zn^2+^/Fe^3+^/Mg-Al-LDH composite was calculated by Equation (9).

(9) $$Removal\;\left(\%\right)=\frac{\left(c_{o}-c_{t}\right)}{c_{o}}\times 100$$

The experimental data of phosphate equilibrium sorption kinetics were fitted to the kinetics model of pseudo-first order (Equation (10)), pseudo-second (Equation (11)), and intraparticle diffusion model (Equation (12)) that considered adsorption might be influenced by diffusion in the spherical adsorbent and by convective diffusion in the phosphate solution.

(10) $$\ln\left(q_{e}-q_{t}\right)=\ln\left(q_{e}\right)-k_{1}t$$

(11) $$\frac{t}{q_{t}}=\frac{1}{k_{2}q_{e}^{2}}+\frac{\;t}{q_{e}}$$

where $k_{1}$ (h^−1^) and $k_{2}$ (g∙mg^−1^∙h^−1^) are the kinetics constants.

(12) qt=kt t12+A

where $k_{t}\;$ (mg∙g^−1^∙h^−1/2^) is the intraparticle diffusion rate constant and A (mg∙g^−1^) is a constant that provides information about the thickness of the boundary layer. The homogenous particle diffusion model was computed for the phosphate sorption onto Mn^2+^/Zn^2+^/Fe^3+^/Mg-Al-LDH composite. If diffusion occurred in the film phase ($D_{f}$, m^2^∙s^−1^) governs the adsorption rate is described by Equation (13), but when the rate of adsorption is controlled by Mn^2+^/Zn^2+^/Fe^3+^/Mg-Al-LDH composite particle diffusion ($D_{p}$, m^2^∙s^−1^) it can be determined by Equation (14) [33].

(13) $$-\ln\left(1-\left(\frac{q_{t}}{q_{e}}\right)\right)=\frac{D_{f}C_{s}}{h\;r\;C_{z}}t$$

(14) $$-\ln\left(1-{\left(\frac{q_{t}}{q_{e}}\right)}^{2}\right)=\frac{2\;\pi^{2}D_{p}}{r^{2}}t$$

where $C_{s}$ (mg∙L^−1^) and $C_{z}$ (mg∙kg^−1^) are the phosphate concentrations in solution and in the Mn^2+^/Zn^2+^/Fe^3+^/Mg-Al-LDH composite, respectively, r is the average radius of the Mn^2+^/Zn^2+^/Fe^3+^/Mg-Al-LDH particle (particles below 200 mesh ≈ radius: 3.7 × 10^−5^ m), t is the contact time (min) and h is the film thickness of the Mn^2+^/Zn^2+^/Fe^3+^/Mg-Al-LDH composite particle (1 × 10^−5^ m for a poorly stirred solution).

#### 2.4.5. Phosphate Adsorption Influenced by Competing Ions

There was a prepared binary combination of phosphate and competing anions, simulating the real wastewater scenery. A measured amount of 0.05 g Mn^2+^/Zn^2+^/Fe^3+^/Mg-Al-LDH composite sample was equilibrated in 25 mL of phosphate solutions containing (25 mg∙L^−1^∙PO_4_^3^^−^ at pH 7.5 which is the expected conditions of treated wastewater). After, it was evaluated the effect on phosphate adsorption by the presence of nitrate, bicarbonate, chloride, and sulfate (25 mg∙L^−1^). The phosphate adsorption also was evaluated in the simultaneous presence of competing anions at the same content level (25 mg∙L^−1^). The essays were performed by triplicate in centrifuge tubes in a rotatory stirrer at 100 rpm at room temperature ~20 °C. The resultant suspension was centrifuged for 10 min and filtered through 0.45 μm cellulose membrane before the determination of phosphate content in the liquid phase.

#### 2.4.6. Desorption of Phosphate Loaded Mn^2+^/Zn^2+^/Fe^3+^/Mg-Al-LDH

A measured amount of 0.05 g Mn^2+^/Zn^2+^/Fe^3+^/Mg-Al-LDH composite sample was equilibrated in 25 mL of phosphate solutions containing (100 mg∙L^−1^∙PO_4_^3^^−^ at pH 7.5 which is the expected conditions of treated wastewater). The essays were performed by triplicate in centrifuge tubes in a rotatory stirrer at 100 rpm at a temperature of ~20 °C. The resultant suspension was centrifuged for 10 min and filtered through 0.45 μm cellulose membrane before the determination of phosphate content in the liquid phase. The solid samples were separated from the aqueous phase for further tests. The saturated phosphate Mn^2+^/Zn^2+^/Fe^3+^/Mg-Al-LDH composite sample was further equilibrated in closed containers using deionized water at pH 3, 6, and 9. The phosphate desorption capacity q_des_ was calculated using Equation (15).

(15) $$q_{des}=\frac{v\times c_{e}}{w}$$

#### 2.4.7. Phosphate Speciation of Loaded Mn^2+^/Zn^2+^/Fe^3+^/Mg-Al-LDH

The species of phosphorus loaded in saturated Mn^2+^/Zn^2+^/Fe^3+^/Mg-Al-LDH composite was determined based on a modified three sequential-step extraction protocol [34]. The labile fraction, metal fraction, and alkaline fractions were quantified. A weighted amount of 0.05 g Mn^2+^/Zn^2+^/Fe^3+^/Mg-Al-LDH composite sample was equilibrated in 25 mL of phosphate solution containing (100 mg∙L^−1^∙PO_4_^3−^ at pH 7.5 which is the expected conditions of treated wastewater). The essay was performed by triplicate in centrifuge tubes in a rotatory stirrer at 100 rpm at room temperature ~20 °C. The resultant suspension was centrifuged for 10 min and filtered through 0.45 μm cellulose membrane before the determination of phosphate content in the liquid phase. The solid samples were separated from the aqueous phase for further tests. The solid sample (Mn^2+^/Zn^2+^/Fe^3+^/Mg-Al-LDH composite) was washed and dried prior to the extraction trials. The loosely bound phosphorus fraction (physical bound) was obtained by two consecutive extractions of Mn^2+^/Zn^2+^/Fe^3+^/Mg-Al-LDH composite sample (0.05 g) in 10 mL of 1 M NH_4_Cl (pH 7). The metal-bound fraction (e.g., iron, aluminum, etc.) was extracted by two consecutive extractions in 10 mL of 0.1 M NaOH followed by extraction in 1 M NaCl. Finally, the phosphorus linked to the alkaline fraction (e.g., calcium, magnesium, etc.) was extracted by two consecutive extractions in 10 mL of 0.5 M HCl. The residual phosphorus (another type of bound) was obtained by means of mass balance between the phosphorus adsorbed and the extracted fractions.

## 3. Results

### 3.1. Characterization

**Figure 1:**
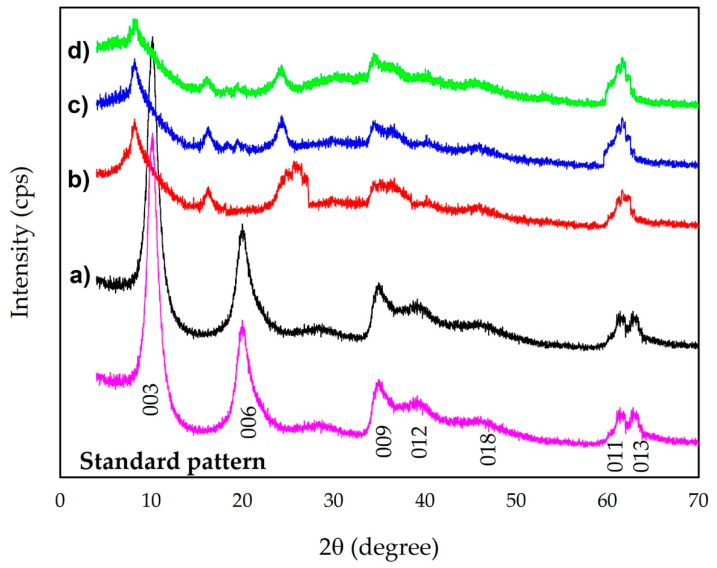
XRD patterns of the LDH materials in comparison with the standard pattern Mg-Al-NO_3_^−^—LDH [20] (**a**) Mg-Al-LDH, (**b**) Mn^2+^/Zn^2+^/Fe^3+^/Mg-Al-LDH composite, (**c**) P-loaded Mn^2+^/Zn^2+^/Fe^3+^/Mg-Al-LDH composite and (**d**) P ions-loaded Mn^2+^/Zn^2+^/Fe^3+^/Mg-Al-LDH composite.

The XRD patterns of Mg-Al-LDH, Mn^2+^/Zn^2+^/Fe^3+^/Mg-Al-LDH composite, and the P-loaded and ions-loaded Mn^2+^/Zn^2+^/Fe^3+^/Mg-Al-LDH composite forms are depicted in comparison with the standard pattern (Figure 1). The diffraction peaks of Mg-Al-LDH match well with the standard (Ref. Code 30000048). The hexagonal crystal hydrotalcite system of parent Mg-Al-LDH was characterized by the lattice parameters a = b = 3.05 Å which is the cation-cation distance in the brucite-like sheet and c = 22.81 Å the thickness of the brucite-like sheet and the interlayer space, comparable with other reported Mg-Al hydrotalcites [17], and indexed in the space group R-3m. The peaks with great intensity were found at 2θ = 10.1° and 20.8° corresponding to the diffraction planes (003) and (006); while the smaller broad and asymmetric peaks were determined at 2θ = 34.8 (009), 39.3 (012), 45.2 (018), 61.3 (110) and 63.2° (113) [35]. The basal spacing d_003_ = 8.74 Å is the sum of the brucite-like sheet (4.80 Å) and the interlayer space (3.94 Å) of the NO_3_^−^ intercalated anions. The Mn^2+^/Zn^2+^/Fe^3+^/Mg-Al-LDH pattern in comparison to the parent Mg-Al hydrotalcite revealed the shifted and change of intensity of some of the characteristic peaks remained in its characteristic form. The occurrence of the anion exchange between the nitrate ions of the parent LDH and the chloride used during the incorporation of Mn^2+^, Fe^3+^, and Zn^2+^. Simultaneously, occurred the precipitation of iron, manganese, and zinc (oxy)hydroxides nanoparticles Fe(OH)_3_ (s), Zn(OH)_2_ (s), and Mn(OH)_2_ (s) by the addition of NaOH (adjusting the pH 9.0) over the surface of Mg-Al-LDH. The incorporation of Mn^2+^, Fe^3+^, and Zn^2+^ resulted in a decrease in the crystallinity as has been reported for other FeMgMn-hydrotalcites [26]. The partial dissolution of the metal hydroxides M(OH)_n_ (s) into the ionic species M^+^ (aq) and OH^−^ (aq) can promote the coexistence of both metal species forms M(OH)_n_ and M^+^. In this study the isomorphic substitution occurred between Mn^2+^, Zn^2+^and Fe^3+^ elements and the original elements (e.g., Mg^2+^ and Al^3+^) of parent Mg-Al-LDH; since Mg^2+^ and Al^3+^ were quantified in the aqueous solution. Besides, the increase of the basal spacing of Mn^2+^/Zn^2+^/Fe^3+^/Mg-Al-LDH composite d_003_ = 9.71 Å was 0.97 Å higher than the parent Mg-Al hydrotalcite (8.74 Å) which is commonly promoted by the increase of cation-cation distance due to the increase of interlayer distance by the decrease of electronegativity of the cations: Fe^3+^ (1.64) > Mn^2+^ (1.60) > Al^3+^ (1.47) according to Allred-Rochow scale. The increase of the lattice parameter of Mn^2+^/Zn^2+^/Fe^3+^/Mg-Al-LDH composite a = b = 3.04 Å and c = 27.7 Å, in comparison to the parent hydrotalcite has been reported as consequence of the isomorphous replacement of Mg^2+^ (0.065 nm) and Al^3+^ (0.053 nm) by higher ionic radius elements such as Fe^3+^ (0.070 nm), Mn^2+^ (0.070 nm) [36]. The Mn-Mg-Fe-Al-LDH after the phosphate and ions adsorption increased to d_003_ = 10.68 Å and d_003_ = 10.70 Å, respectively. The increase of the basal spacing in comparison with the non-adsorbed Mn^2+^/Zn^2+^/Fe^3+^/Mg-Al-LDH composite was attributed to the phosphate and anions intercalation in the interlayer.

**Figure 2:**
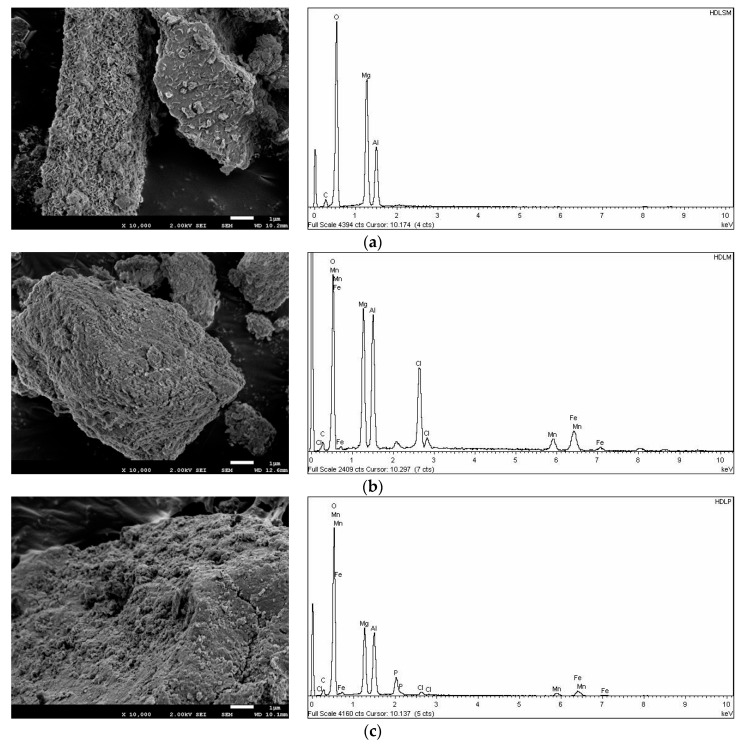
SEM–EXD of the LDH materials (**a**) Mg-Al-LDH, (**b**) Mn^2+^/Zn^2+^/Fe^3+^/Mg-Al-LDH composite and (**c**) P-loaded Mn^2+^/Zn^2+^/Fe^3+^/Mg-Al-LDH composite.

The SEM images of the Mg-Al-LDH showed the typical structure of LDH (Figure 2). A layered morphology was observed with plate-like particles with an average size of 0.25–0.5 µm which is in accordance with other reports [17]. After, the incorporation of Mn^2+^, Fe^3+^, and Zn^2+^ on the LDH small particles covering the platelet form of parent Mg-Al-LDH was observed. The morphology of the Mn^2+^/Zn^2+^/Fe^3+^/Mg-Al-LDH composite obtained in this study differs from the morphology of metal LDHs materials obtained in a single-step co-precipitation method [9]. It demonstrated the adsorbent developed in this study has different nature in comparison to the rest of the LDHs materials previously reported. The FeMgMn-LDH obtained for phosphate adsorption capacity demonstrate a totally smooth, plate-like morphology [26]. We attribute the precipitation of iron, manganese, and zinc (oxy)hydroxides nanoparticles that occurred over the parent Mg-Al-LDH, as was expected. The surface of the Mn^2+^/Zn^2+^/Fe^3+^/Mg-Al-LDH composite got rougher after the phosphate adsorption because small particles with platelet form covered the surface. This morphology of the phosphate-doped Mn^2+^/Zn^2+^/Fe^3+^/Mg-Al-LDH is comparable with the saturated FeMgMn-LDH [26]. Further assays will determine the effectiveness and behavior of the developed Mn^2+^/Zn^2+^/Fe^3+^/Mg-Al-LDH composite in comparison with other LDHs materials.

The semiquantitative results of Mg-Al-LDH and Mn^2+^/Zn^2+^/Fe^3+^/Mg-Al-LDH composite are summarized in Table 1. The parent Mg-Al-LDH was characterized by the presence of magnesium, aluminum, and oxygen. After the incorporation of Mn^2+^/Zn^2+^/Fe^3+^ in the parent Mg-Al-LDH, it was determined the presence of new elements (e.g., chloride, manganese, zinc, and iron). The reduction of Mg^2+^ and Al^3+^ from parent Mg-Al-LDH was also verified by quantification in an aqueous solution with Mg^2+^: 4 mg∙L^−1^ and Al^3+^: 8 mg∙L^−1^. The above-mentioned mechanism of isomorphic substitution between Mn^2+^, Zn^2+^, and Fe^3+^ and both Mg^2+^ and Al^3+^ from the parent Mg-Al-LDH was confirmed. The existence of chloride in the Mn^2+^/Zn^2+^/Fe^3+^/Mg-Al-LDH only can be explained in terms of anion exchange between chloride and nitrate from the parent Mg-Al-LDH, because in aqueous solution it was determined 14 mg∙L^−1^∙NO_3_^−^. After the phosphate adsorption, the reduction of the chloride content occurred due to the anions exchange between phosphate and both chloride and nitrate from the interlayer of Mn^2+^/Zn^2+^/Fe^3+^/Mg-Al-LDH composite (the aqueous solution content was determined as NO_3_^−^: 4 mg∙L^−1^ and Cl^−^: 5 mg∙L^−1^). Previous reports established metals cations (e.g., Mn^2+^, Zn^2+^, and Fe^3+^) are incorporated into the LDH structure by means of specific adsorption [inner sphere complexation reactions with the surface functional hydroxyl groups (OH^−^)] and by non-specific adsorption [outer sphere electrostatic interactions due to the existence of deprotonated hydroxyl groups (-O^−^)] [9]. Thus, the incorporation of Mn^2+^/Zn^2+^/Fe^3+^ (oxy)hydroxide nanoparticles onto the Mg-Al-LDH composite were performed by precipitation, isomorphic substitution, and surface complexation.

The specific surface area of parent Mg-Al-LDH was measured as 49.1 m^2^∙g^−1^ was higher than the functionalized Mn^2+^/Zn^2+^/Fe^3+^/Mg-Al-LDH composite with a value of 37.8 m^2^∙g^−1^. The decrease in surface area was attributed to the intercalation of metals and this behavior was comparable with other hydrotalcites [35]. Even though our developed Mn^2+^/Zn^2+^/Fe^3+^-Mg-Al-LDH demonstrated a lower surface area in comparison to the parent Mg-Al-LDH. The phosphate adsorption onto LDHs has been reported not to be dependent at all on the surface area since other mechanisms are also involved [22].

**Figure 3:**
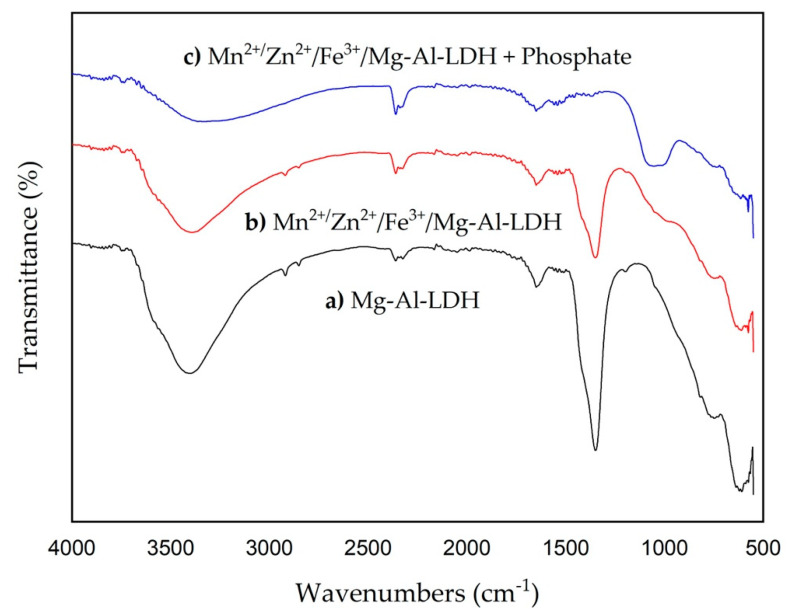
FTIR spectra of the LDH materials (**a**) Mg-Al-LDH, (**b**) Mn^2+^/Zn^2+^/Fe^3+^/Mg-Al-LDH composite, and (**c**) P-loaded Mn^2+^/Zn^2+^/Fe^3+^/Mg-Al-LDH composite.

The functional groups at the surface of parent Mg-Al-LDH and Mn^2+^/Zn^2+^/Fe^3+^/Mg-Al-LDH composite were determined by Fourier transform analysis. The FTIR spectra of both LDH samples are depicted in Figure 3. The FTIR spectrum of the parent Mg-Al-LDH revealed four main bands at 3398, 1649, 1348, 748, and 608 cm^−1^. The broad peak at 3398 cm^−1^ belongs to the stretching vibration of -OH groups from brucite-like layers and interlayer water molecules. The band at 1348 cm^−1^ is related to the NO_3_^−^ groups existing in the interlayer of the hydrotalcite materials [35]. Besides, the bands at 608 and 748 cm^−1^ are attributed to the metal (M: Mg and Al)-oxygen bands (e.g., M-O stretching and M-O-H bending) and the band at 1649 cm^−1^ belongs to the water bending vibration of interlayer water [17]. The FTIR spectra almost remained constant after the incorporation of metal cations (e.g., Mn^2+^, Zn^2+^, and Fe^3+^) on the parent Mg-Al-LDH. However, the main change was identified at 3398 cm^−1^ which belongs to the -OH groups, the shift of this band was attributed to the incorporation of Mn^2+^, Zn^2+^, and Fe^3+^ cations on the Mn^2+^/Zn^2+^/Fe^3+^/Mg-Al-LDH composite structure. After, the phosphate adsorption again the shift of the band at 3398 cm^−1^ was associated with the involvement of the -OH groups with the phosphate removal from synthetic wastewater. After phosphate adsorption, the band at 1348 cm^−1^ shifted and the appearance of a new band at 1054 cm^−1^ was attributed to the phosphate incorporation on the Mn^2+^/Zn^2+^/Fe^3+^/Mg-Al-LDH composite structure.

The point of zero charge of the parent Mg-Al-LDH was found to be pH_PZC_ 9.6 while the functionalized Mn^2+^/Zn^2+^/Fe^3+^/Mg-Al-LDH composite was pH_PZC_ 8.9. The decrease of the pH_PZC_ of Mn^2+^/Zn^2+^/Fe^3+^/Mg-Al-LDH composite in comparison with the parent Mg-Al-LDH suggested some changes occurred. The shift of the pH_PZC_ has been associated with the formation of M-OH groups (e.g., FeOH, ZnOH, MnOH) over the surface of Mn^2+^/Zn^2+^/Fe^3+^/Mg-Al-LDH composite. The hydroxylation process occurs conventionally when the existing metal cations (e.g., Mn^2+^, Zn^2+^, and Fe^3+^) on the surface of the Mn^2+^/Zn^2+^/Fe^3+^/Mg-Al-LDH composite is exposed to water, which promotes coordination with hydroxyl groups.

### 3.2. Effect of pH

**Figure 4:**
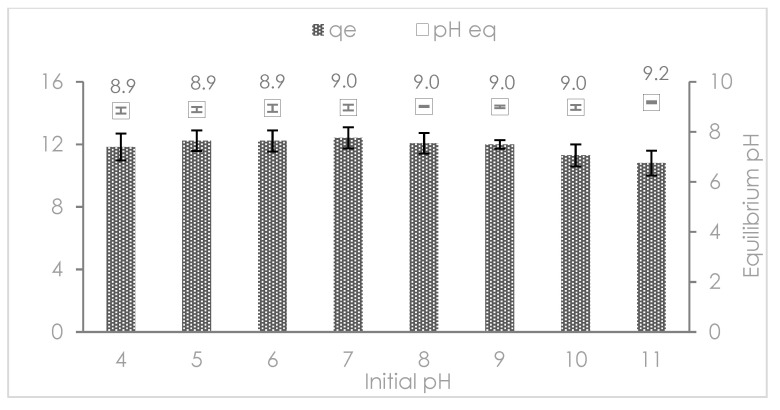
Phosphate adsorption capacity as a function of the pH and equilibrium pH.

The phosphate adsorption as a function of the pH solution is depicted in Figure 4. For a better understanding of the adsorption mechanisms, it must be also considered the distribution of the orthophosphate species in solution by the effect of the pH (Equation (16)).

(16) $$H_{3}{\mathrm{PO}}_{4}⇌H_{2}{\mathrm{PO}}_{4}^{-}{\;\mathrm{pK}}_{a}=2.1\;H_{2}{\mathrm{PO}}_{4}^{-}\;⇌{\mathrm{HPO}}_{4}^{2-}{\;\mathrm{pK}}_{a}=7.20\;{\mathrm{HPO}}_{4}^{2-}⇌{\mathrm{PO}}_{4}^{3-}{\;\mathrm{pK}}_{a}=12.35$$

The phosphate adsorption capacity remained invariable in the pH range from 4 to 9 which is below the pH_PZC_ 8.9 ± 0.1 of Mn^2+^/Zn^2+^/Fe^3+^/Mg-Al-LDH composite. The phosphate adsorption can be explained in terms of electrostatic attraction between both H_2_PO_4_^−^ and HPO_4_^2−^ species and the positive charge of the electric field over the surface of Mn^2+^/Zn^2+^/Fe^3+^/Mg-Al-LDH composite (pH < pH_PZC_) due to the protonation of metal cations hydroxyl groups. The formed sites are very reactive for phosphate removal due to hydrogen bonding interactions. Phosphate anions (especially, HPO_4_^2^^−^) have high basicity with a pair of high electronic densities that can form a hydrogen bond with the protonated Mn^2+^/Zn^2+^/Fe^3+^/Mg-Al-LDH composite surface -(OH)^+^ [37,38]. However, it was not detected any new mineral phase by means of XRD analysis. On the other hand, the decrease of phosphate adsorption capacity occurred at pH 10 and 11 (pH > pH_PZC_) which is attributed to the hydroxylation of metal cations hydroxyl groups and the hard Lewis base (OH^−^ ions) [39] at the surface of the Mn^2+^/Zn^2+^/Fe^3+^/Mg-Al-LDH composite promoting the electrostatic repulsion effect with the phosphate H_2_PO_4_^−^, HPO_4_^2−^ and PO_4_^3−^ species. Even though above the pH_PZC_ > 8.9 ± 0.1 the Mn^2+^/Zn^2+^/Fe^3+^/Mg-Al-LDH composite is supposed to promote the electrostatic repulsion effect canceling the phosphate adsorption. However, the slight reduction of the phosphate adsorption capacity above the pH_PZC_ suggested that physical adsorption is not the unique governing mechanism. We detect a low content of Fe^3+^, Mn^2+^, Zn^2+^, Mg^2+^ and Al^3^^+^ in aqueous solution after the phosphate adsorption by means of ionic chromatography (Fe^3+^: 4 mg∙L^−1^, Mn^2+^: 2 mg∙L^−1^, Zn^2+^: 2 mg∙L^−1^, Mg^2+^: 2 mg∙L^−1^ and Al^3+^: 4 mg∙L^−1^ at pH 7.5). Besides, the semiquantitative analysis of Mn^2+^/Zn^2+^/Fe^3+^/Mg-Al-LDH (Table 1), also suggested the reduction of Fe^3+^, Mn^2+^, Zn^2+^, Mg^2+,^ and Al^3+^ content after the phosphate adsorption. The release of Fe^3+^, Mn^2+^, Zn^2+^, Mg^2+,^ and Al^3+^ cations after the phosphate adsorption was in accordance with the conventional behavior of LDHs materials. The removal of phosphate has been reported to occur in three steps: (i) the phosphate complexation with the surface hydroxide, (ii) the adsorbed phosphate acts as a new adsorption site for the dissolved metal ions hydroxide and (iii) the adsorbed metal ion hydroxide act as a new sorption site for the phosphate remaining in the solution [40]. In the conditions used in this study, the phosphate complexation occurred when the phosphate replaced the protonated hydroxyl groups generating mono-dentate and bi-dentate inner sphere phosphate complexes with the release of OH^−^ as a secondary product. The released cations from the LDH structure, as well as from the partial dissolution of the metal Mn^2+^/Zn^2+^/Fe^3+^ (oxy)hydroxides, supported the LDH occurrence. Hence, the important issue of the pH of the equilibrated solution after the phosphate adsorption that reached an average value of 8.9 ± 0.1 (Figure 4) which is associated with the buffering pH function of metal cations (e.g., Mn^2+^, Zn^2+^, and Fe^3+^) and their hydroxides [37]. The hydroxide forms of Mn^2+^/Zn^2+^/Fe^3+^ released metals precipitated over the adsorbed phosphate. The Mn^2+^/Zn^2+^/Fe^3+^ metal ion hydroxides act as new sorption sites for the phosphate remaining in the solution. The released cations and their hydroxides work effectively as coagulants/or precipitants for phosphate removal on the surface [41] which is another mechanism of adsorption enhancing phosphate removal. Thus, the existence of Fe^3+^, Mn^2+^, Zn^2+^, Mg^2+^, and Al^3+^ in an aqueous solution can be explained in terms of the residual fractions of metal cations that cannot be precipitated in the hydroxide or phosphate forms [41]. The FTIR analysis revealed the participation of the OH groups of Mn^2+^/Zn^2+^/Fe^3+^/Mg-Al-LDH composite during the phosphate adsorption. Our theory about the phosphate complexation reactions was endorsed by the increase of the equilibrium pH by the effect of the release of OH^−^ as a secondary product. Finally, anion exchange also occurred between both NO_3_^−^ and Cl^−^ the interlayer anions of Mn^2+^/Zn^2+^/Fe^3+^/Mg-Al-LDH composite and both phosphate H_2_PO_4_^−^ and HPO_4_^2−^ species since the release of NO_3_^−^ (4 mg∙L^−1^) and Cl^−^ (5 mg∙L^−1^) anions were verified by means of ionic chromatography.

The Mn^2+^/Zn^2+^/Fe^3+^/Mg-Al-LDH used in this study has an advantage in comparison to other functional materials because of the high effectiveness of phosphate removal at real pH conditions of treated wastewater (between pH 6 and 8). The use of Mn^2+^/Zn^2+^/Fe^3+^/Mg-Al-LDH composite can be an efficient alternative for the phosphate recovery from wastewater treatment plants at their operational conditions pH of main- or side-streams after anaerobic digestion [42].

### 3.3. Phosphate Adsorption Isotherms

The phosphate adsorption onto Mn^2+^/Zn^2+^/Fe^3+^/Mg-Al-LDH composite was fitted to the Langmuir and Freundlich isotherms models (Table 2). The maximum adsorption capacity of an adsorbent is one of the most important physicochemical parameters to characterize the adsorbent’s performance [43]. In this study, we select the Langmuir and Freundlich isotherms models as practical guidelines for the description of phosphate adsorption data. Even though they cannot be used to prove a specific sorption mechanism (e.g., precipitation, ion exchange, etc.) [30]. Our results can be compared to other reported LDH materials that used Langmuir and Freundlich isotherm models. For example, the values of phosphate maximum adsorption capacities developed by our Mn^2+^/Zn^2+^/Fe^3+^/Mg-Al-LDH composite in this study are around 80 mg∙g^−1^, which is higher than other reported for LDHs materials obtained by a single step co-precipitation method and other adsorbents previous reported (Table 2).

The adsorption capacity is one of the strengths of using the Mn^2+^/Zn^2+^/Fe^3+^/Mg-Al-LDH composite obtained by the incorporation of metals (oxy)hydroxide nanoparticles onto a parent Mg-Al-LDH in comparison to the metal LDHs adsorbents conventionally reported obtained by the synthesis in a single co-precipitation step. The higher phosphate adsorption capacity developed by the Mn^2+^/Zn^2+^/Fe^3+^/Mg-Al-LDH composite demonstrated the metal (oxy)hydroxide nanoparticles potentiate the sorption characteristics of the individual LDHs materials. The incorporation of metal hydroxides (e.g., Mn^2+^, Zn^2+^, and Fe^3+^) seems to be practical for the potential use of this material at full scale since the particle size problem of this hydroxides can be solved by supporting in a stable template such as LDHs. Even though our prepared Mn^2+^/Zn^2+^/Fe^3+^/Mg-Al-LDH composite has different physicochemical characteristics compared with the conventionally LDHs it becomes efficient. It is in accordance with previous reports about phosphate adsorption onto LDHs and is partially governed by surface adsorption [22]. Other mechanisms are involved in phosphate adsorption which will be discussed in the following lines. The Mn^2+^/Zn^2+^/Fe^3+^/Mg-Al-LDH turned out to be a [26] sensitive phosphate adsorbent due to its effectiveness in a broad range of phosphate concentrations. Similar behavior has been reported for other sorbents where the increase in phosphate concentration promotes a higher driving force promoting an easier mass transfer to the sorbent surface [38]. The experimental data of phosphate adsorption were best fitted to the Langmuir isotherm model at the different temperatures evaluated in this study with R^2^ values ~1. Then, the phosphate adsorption can be explained in terms of chemical adsorption in monolayer, so phosphate adsorption occurred at specific sites on the surface of Mn^2+^/Zn^2+^/Fe^3+^/Mg-Al-LDH composite. It validates the above-discussed mechanisms of phosphate adsorption (e.g., complexation, precipitation, and anion exchange). Since a homogeneous surface characterized the Mn^2+^/Zn^2+^/Fe^3+^/Mg-Al-LDH composite in this study the phosphate adsorption is supposed not to be at all well described by the Freundlich model [38]. The physical adsorption by means of hydrogen bonding is one of the multiple mechanisms that promoted the phosphate adsorption onto the Mn^2+^/Zn^2+^/Fe^3+^/Mg-Al-LDH composite. The theory about the phosphate complexation reactions is endorsed by the increase of the equilibrium pH by the effect of the release of OH^−^ as a secondary product. The formation of monodentate and bidentate inner sphere complexes during phosphate adsorption is described by Equation (17).



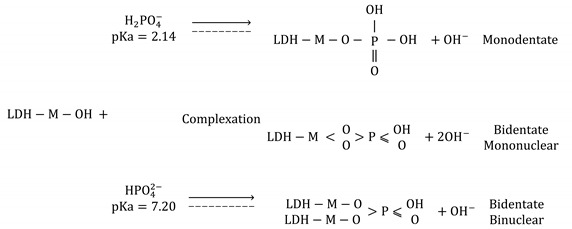

(17)


**Figure 5:**
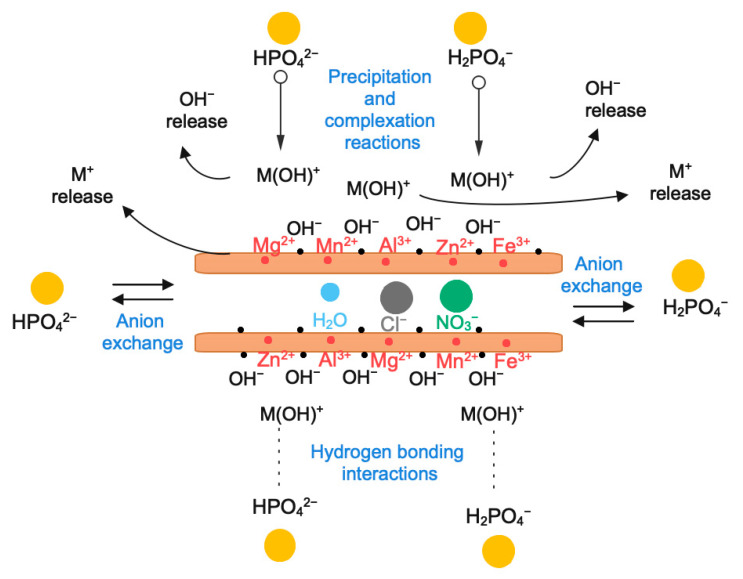
Phosphate adsorption mechanisms onto Mn^2+^/Zn^2+^/Fe^3+^/Mg-Al-LDH composite at pH 7.5.

A summary of the mechanism of phosphate adsorption onto Mn^2+^/Zn^2+^/Fe^3+^/Mg-Al-LDH above discussed is presented in Figure 5. Therefore, phosphate adsorption was promoted by both physical and chemical mechanisms. Four mechanisms were determined to occur during phosphate adsorption: hydrogen bonding, complexation reactions, precipitation, and anion exchange. The hydrogen bonding adsorption mechanism was established considering the point of zero charge. The phosphate adsorption increased below the pH_PZC_ which is the region of positive charges over the surface of Mn^2+^/Zn^2+^/Fe^3+^/Mg-Al-LDH. It was discarded the hypothesis of the uniqueness of hydrogen bonding as a phosphate adsorption mechanism since the pH_PZC_ was important when it was expected to be null. The phosphate complexation, as a second mechanism, occurred when the phosphate replaced the protonated hydroxyl groups generating mono-dentate and bi-dentate inner sphere phosphate complexes. The adsorbed phosphate becomes a new adsorption site for the dissolved metal ions released from the LDHs structure as well as from the partial dissolution of the metal Mn^2+^/Zn^2+^/Fe^3+^ (oxy)hydroxides supported in the LDH. The release of OH^−^ as a secondary product of the phosphate complexation reaction, promotes the formation of the hydroxides forms of Mn^2+^/Zn^2+^/Fe^3+^ released metals which precipitate over the adsorbed phosphate. The Mn^2+^/Zn^2+^/Fe^3+^ metal ion hydroxides act as new sorption sites for the phosphate remaining in the solution. The precipitation of phosphate is the third mechanism of removal; being the supported Mn^2+^/Zn^2+^/Fe^3+^ (oxy)hydroxides determinant for the enhancement of phosphate adsorption. The anion exchange as the fourth mechanism involved in phosphate adsorption was deduced from the chloride and nitrate anions released in the aqueous solution. The release of low contents of Mn^2+^/Zn^2+^/Fe^3+^ into water is part of the precipitation mechanisms associated with phosphate adsorption rather than the adsorbent instability. A piece of relevant information about the phosphate adsorption onto Mn^2+^/Zn^2+^/Fe^3+^/Mg-Al-LDH composite is that effectiveness does not depend at all on the crystallinity, morphology, and surface area because the surface adsorption is not the unique mechanism [22]. Even though the Mn^2+^/Zn^2+^/Fe^3+^/Mg-Al-LDH composite has a relatively low crystallinity, low surface area, and a rougher morphology in comparison to its parent Mg-Al-LDH it was very efficient for phosphate removal.

**Figure 6:**
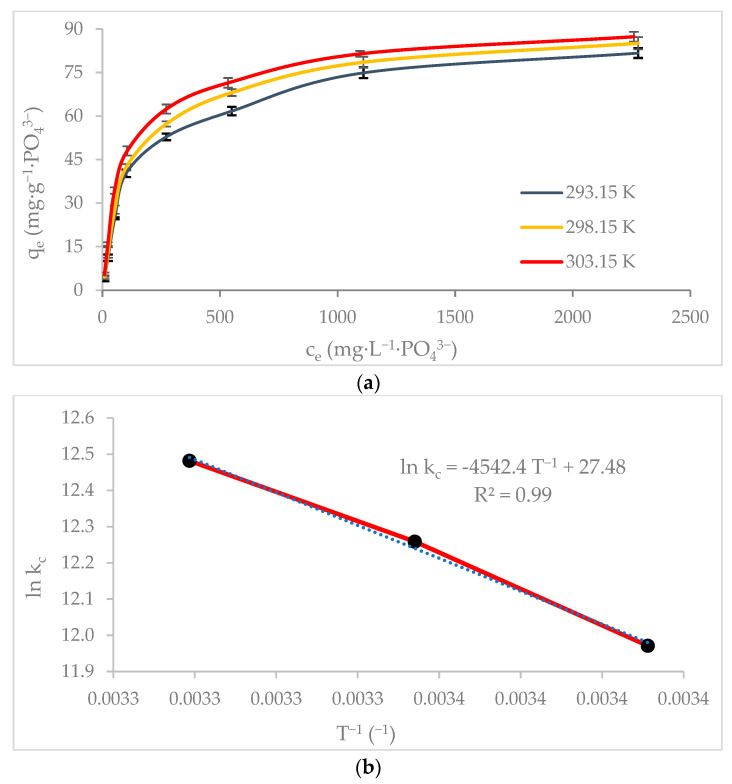
Equilibrium adsorption data: (**a**) isotherms as a function of the temperature and (**b**) thermodynamic study of phosphate adsorption onto Mn^2+^/Zn^2+^/Fe^3+^/Mg-Al-LDH composite.

The phosphate adsorption isotherms at the different temperatures used in this study are depicted in Figure 6. The adsorption onto Mn^2+^/Zn^2+^/Fe^3+^/Mg-Al-LDH is characterized by the high adsorption capacities reached at low concentrations; however as high concentrations have reached the saturation of the adsorbent is achieved. The temperature also promotes the increase of adsorption capacity, especially the effect of temperature is important at high concentrations.

The values of maximum phosphate adsorption capacities by Mn^2+^/Zn^2+^/Fe^3+^/Mg-Al-LDH composite increased above 2% with the increase of the system temperature at 293.15, 298.15, and 303.15 K (Table 3). At the three temperatures evaluated the experimental data were best fitted to the Langmuir isotherm model and the $r_{L}$ values were between 0 and 1, demonstrating the phosphate adsorption onto Mn^2+^/Zn^2+^/Fe^3+^/Mg-Al-LDH composite was favorable at these conditions.

Also, the thermodynamic parameters such as Gibbs free energy (∆G^0^), entropy (∆S^0^), and enthalpy (∆H^0^) were used for describing the phosphate adsorption (Table 4). The results obtained in this study are similar to those reported for a FeMg2Mn-LDH where the positive value of ∆H^0^ = 37.77 kJ·mol^−^^1^ denote that the phosphate adsorption onto Mn-Mg-Fe-Al-LDH was endothermic and the negative values of ∆G^0^ = −29.18, −30.39 and −31.46 kJ.mol^−1^ indicates a decrease of spontaneity at higher temperatures. Besides, the positive value of (∆S**^0^**) = 0.23 kJ·mol^−1^·K^−1^ represent the increase of the disorder at the interface of the solid–solutions system [26]. The ∆G**^0^** values below −20 kJ·mol^−1^ revealed that surface complexation is the main mechanism of phosphate adsorption with the substantial contribution of electrostatic interaction [47].

### 3.4. Phosphate Adsorption Kinetics

**Figure 7:**
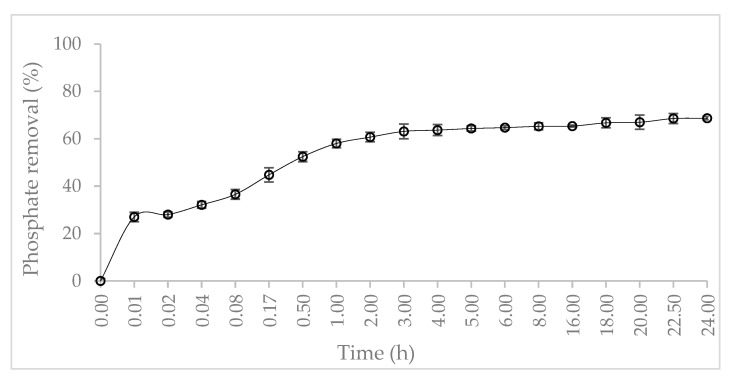
Phosphate removal profile onto Mn^2+^/Zn^2+^/Fe^3+^/Mg-Al-LDH as a function of time.

The phosphate removal by Mn^2+^/Zn^2+^/Fe^3+^/Mg-Al-LDH composite as a function of time is depicted in Figure 7. 50% of phosphate was removed from the synthetic wastewater solution within the five initial minutes. Within one hour the phosphate removal increased to 80%, at this time the equilibrium adsorption was almost reached. The fast adsorption of phosphate from synthetic aqueous solution is suitable for the application of Mn^2+^/Zn^2+^/Fe^3+^/Mg-Al-LDH composite in stirred reactors. The occurrence of electrostatic interaction and anion exchange mechanism seems to be responsible for the fast phosphate adsorption at the initial stage. Since phosphate complexation and precipitation are chemical processes that have higher time and energy requirements. The performance of phosphate adsorption onto Mn^2+^/Zn^2+^/Fe^3+^/Mg-Al-LDH composite is better than other adsorbents used for this purpose (e.g., natural zeolites, natural clays) [2,46].

The phosphate adsorption kinetic parameters are summarized in Table 5. The experimental data of phosphate adsorption was best fitted to the pseudo-second-order kinetic model and an R^2^ value ≈ 0.99 was obtained. The pseudo-second-order kinetic model attributed to the occurrence of chemical adsorption is in concordance with the phosphate complexation reactions above discussed. The experimental data was also well described by the intraparticle diffusion model. The intraparticle diffusion was not the unique rate-limiting step during phosphate adsorption since the plot revealed the existence of multi-stage adsorption. The phosphate adsorption onto Mn^2+^/Zn^2+^/Fe^3+^/Mg-Al-LDH composite was characterized by three specific phases: an initial stage of fast rate, followed by a slower rate, and a final equilibrium stage. The first stage was promoted by the film diffusion of phosphate through the hydrodynamic layer and then diffusion through the boundary layer to the external surface of the Mn^2+^/Zn^2+^/Fe^3+^/Mg-Al-LDH composite. The second stage when the rate slows down the adsorption was endorsed by intraparticle diffusion. The final equilibrium phase was characterized by the decrease of phosphate concentration in the synthetic wastewater solution and the reduction of active sites on Mn^2+^/Zn^2+^/Fe^3+^/Mg-Al-LDH composite [47]. The values of effective diffusion coefficients (D_p_ and D_f_) were determined in the range of 2.6 × 10^−14^ and 5.6 × 10^−15^ m^2^·s^−1^, respectively. The diffusion coefficients in this study are lower in comparison to other phosphate adsorbents such as metallic-loaded zeolites and clays (e.g., Mn^2+^, Zn^2+^, and Fe^3+^) since no comparable information was easily found on LDH materials.

### 3.5. Influence of Competing Ions

The effect of competing ions on the phosphate adsorption onto the Mn^2+^/Zn^2+^/Fe^3+^/Mg-Al-LDH composite is summarized in Table 6. The absence of competing anions (q^0^) was compared with the presence of each anion (q^mix^) and quantified as the adsorption ratio (q^mix^/q^0^) determining the decrease or improvement of phosphate adsorption. The phosphate adsorption capacity onto the Mn^2+^/Zn^2+^/Fe^3+^/Mg-Al-LDH composite was reduced a 10% by the presence of sulfate. In the simultaneous presence of competing anions (e.g., nitrate, sulfate, chloride, bicarbonate) a reduction of 20% of phosphate capacity was obtained. The inhibition of phosphate adsorption in presence of sulfate is explained in terms of preference for divalent charge density anions in comparison to monovalent anions as it has been previously reported for LDH materials [48]. The interference in the phosphate adsorption promoted by the coexisting anionic species (nitrate, sulfate, chloride, bicarbonate) has been attributed to the occupation of specific bonding sites [49]. On the other hand, the phosphate adsorption capacity was maintained in the presence of chloride; this phenomenon can be explained due to the preference of LDH materials towards divalent charge density anions such as phosphate. Also, chloride has been reported not to be a good competitor for phosphate adsorption since it forms preferably outer sphere complexes [47] verifying that monovalent anions did not interfere in the divalent anions adsorption. The phosphate adsorption capacity in presence of bicarbonate and nitrate was noteworthily potentiated in 10% and 40%, respectively [47]. The increase of phosphate adsorption on Mn^2+^/Zn^2+^/Fe^3+^/Mg-Al-LDH composite by the effect of nitrate and bicarbonate is explained in terms of the increase of ionic strength in the solution [41]. The existence of new mineralogical phases after the phosphate adsorption on Mn^2+^/Zn^2+^/Fe^3+^/Mg-Al-LDH composite in presence of competing ions was discarded by means of XDR analysis maybe be due to the content below the detection limit.

The Mn^2+^/Zn^2+^/Fe^3+^/Mg-Al-LDH composite was selective towards phosphate adsorption in presence of coexisting anions. The selectivity of Mn^2+^/Zn^2+^/Fe^3+^/Mg-Al-LDH becomes an important operational feature since wastewater composition is diverse. Mn^2+^/Zn^2+^/Fe^3+^/Mg-Al-LDH becomes a promising material for further application in wastewater treatment processing plants for phosphate recovery purposes.

### 3.6. Phosphate Speciation

The phosphate chemical forms extracted from loaded Mn^2+^/Zn^2+^/Fe^3+^/Mg-Al-LDH composite are summarized in Table 7. The value of loosely bound phosphorus fraction (LB-P) was 14 ± 1% which is the phosphorus immobilized by means of physical adsorption and it is available for plants. It validates our hypothesis of electrostatic attraction (outer sphere complexation) is not the unique mechanism involved in phosphate adsorption. Besides, the loosely bound phosphorus fraction provides a guideline about the availability of phosphorous for soils and plants in the scenery of further use. The phosphorous bounded to metallic species (e.g., Mn^2+^, Zn^2+^, and Fe^3+^) hydroxides that belong to the (Fe + Zn + Mn)–P fraction is equal to 51%. It suggests the participation of hydrated metal oxide groups in phosphate adsorption by means of inner sphere complexation and precipitation. Additionally, the phosphorous bound to magnesium and other alkaline metals belong to the (Mg)–P fraction. It suggests the phosphate is immobilized by the participation of precipitation reactions with the Mg hydroxides, even though no new mineralogical phases were identified by means of XRD analysis. Information about phosphate LDH fractioning was not easily obtained for comparison in this section.

### 3.7. Adsorption–Desorption Cycles

**Figure 8:**
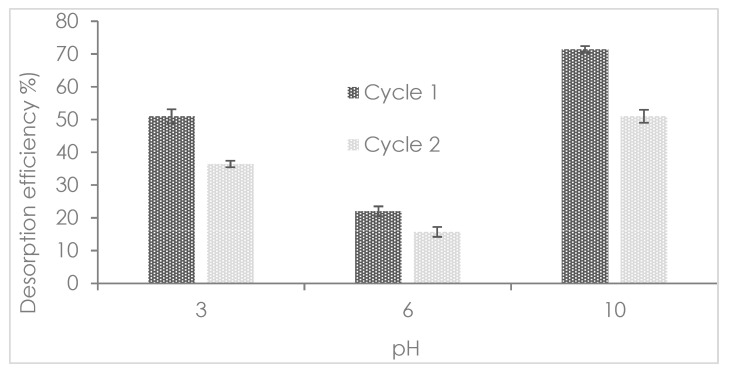
Phosphate adsorption-desorption profile as a function of pH at two cycles.

The phosphate adsorption-desorption profile in two cycles is depicted in Figure 8. The optimal condition for desorption of adsorbed phosphate onto Mn^2+^/Zn^2+^/Fe^3+^/Mg-Al-LDH composite was at a basic pH value equal to 10 becoming over 70% of the recovery. The second cycle of desorption reached over 50% recovery validating the optimal conditions for desorption at pH 10. The results of regeneration confirmed the phosphate adsorption onto Mn^2+^/Zn^2+^/Fe^3+^/Mg-Al-LDH composite was governed by means of physical adsorption as well as chemical adsorption. At pH 10 the phosphate (HPO_4_^2−^) could be recovered due to the total physical adsorption reversibility and the partial chemical adsorption reversibility at it has been reported for other phosphate adsorbents [3]. The desorption reached 50% in the first cycle and 35% in the second cycle in the desorption essay at pH 3. At this pH the destruction of the LDH structure has been reported, so higher amounts of Mg^2+^ in an aqueous solution can promote the phosphate (e.g., H_2_PO_4_^−^ and HPO_4_^2^^−^) complexation and precipitation reactions avoiding the phosphate release. At acid conditions, the phosphate bound to metal (e.g., Mn^2+^, Zn^2+^, and Fe^3+^) hydroxyl groups can be partially desorbed [2]. The lowest phosphate desorption capability of Mn^2+^/Zn^2+^/Fe^3+^/Mg-Al-LDH composite was determined to be at pH 6. The phosphate anionic form (H_2_PO_4_^−^) was the main specie recovered from the Mn^2+^/Zn^2+^/Fe^3+^/Mg-Al-LDH composite. At this condition it was demonstrated the immobilized phosphate by chemical adsorption cannot be desorbed and only the physical adsorbed phosphate was recovered. The Mn^2+^/Zn^2+^/Fe^3+^/Mg-Al-LDH composite can be used in several cycles of phosphate adsorption-desorption however it has limited reusability with a reduction of efficiency after each cycle.

The limited reusability of Mn^2+^/Zn^2+^/Fe^3+^/Mg-Al-LDH composite becomes a new alternative for the environmentally friendly final disposal of the adsorbent, which is in accordance with the conception of our study. The Mn^2+^/Zn^2+^/Fe^3+^/Mg-Al-LDH composite can be used in two consecutive cycles of adsorption-desorption and after disposal as soil amendment material. The exhausted Mn^2+^/Zn^2+^/Fe^3+^/Mg-Al-LDH is an integral system of macro and micronutrients (P, Fe, Mn, Zn) that would improve the physical and chemical properties of the soil. Besides, the high selectivity of Mn^2+^/Zn^2+^/Fe^3+^/Mg-Al-LDH towards phosphate elucidate the viability of using this material for agricultural purpose since no harmful pollutants can be released into soil and water.

## 4. Conclusions

In this study, Mn^2+^, Zn^2+^, and Fe^3+^ (oxy)hydroxide nanoparticles were supported onto a parent Mg-Al-LDH obtaining the Mn^2+^/Zn^2+^/Fe^3+^/Mg-Al-LDH composite for the phosphate recovery from simulated urban treated wastewater. The Mn^2+^/Zn^2+^/Fe^3+^/Mg-Al-LDH composite revealed a high capacity and good efficiency for phosphate adsorption at the pH range of real treated wastewater which is an improvement in comparison with other adsorbents used for this purpose. The Mn^2+^/Zn^2+^/Fe^3+^/Mg-Al-LDH composite demonstrates higher adsorption capacities in comparison to the metal LDH synthesized in a single co-precipitation method. The phosphate adsorption was strongly potentiated by the effect of Mn^2+^, Zn^2+^, and Fe^3+^ (oxy)hydroxide nanoparticles. The phosphate immobilization onto Mn^2+^/Zn^2+^/Fe^3+^/Mg-Al-LDH composite was promoted by the simultaneous occurrence of physical and chemical adsorption. The chemical complexation, anion exchange, electrostatic attraction, and precipitation were the main mechanisms that governed the phosphate adsorption onto Mn^2+^/Zn^2+^/Fe^3+^/Mg-Al-LDH composite. The spontaneous and endothermic behavior was determined for phosphate adsorption onto Mn^2+^/Zn^2+^/Fe^3+^/Mg-Al-LDH composite. The intraparticular diffusion model described the best kinetical data due to the existence of three well-defined stages of adsorption. The presence of sulfate and chloride was irrelevant for the phosphate adsorption since they did not interfere with the ability of phosphate to adsorb in the specific and non-specific bonding sites of Mn^2+^/Zn^2+^/Fe^3+^/Mg-Al-LDH composite. The phosphate adsorption was potentiated by nitrate and bicarbonate due to the increase of ionic strength in the solution. The reusability of Mn^2+^/Zn^2+^/Fe^3+^/Mg-Al-LDH composite is limited to two cycles of operation which suppose a weakness in comparison to polymeric exchangers. However, the saturated phosphate solutions obtained from adsorbent regeneration can be used for soil amendment application. Then, the use of Mn^2+^/Zn^2+^/Fe^3+^/Mg-Al-LDH composite in tertiary wastewater treatment could reduce the phosphorous contents within the normative and being a new source of phosphorous nutrients for agriculture. The exhausted Mn^2+^/Zn^2+^/Fe^3+^/Mg-Al-LDH composite could be finally disposed of for soil amendment since it is highly selective towards phosphate and did not represent an environmental problem with the release of harmful pollutants.

## Figures and Tables

**Table 1:** Semiquantitative elemental composition of LDH materials.

Material	C (%)	O (%)	Mg (%)	Al (%)	P (%)	Cl (%)	Mn (%)	Fe (%)	Zn (%)
Mg-Al-LDH	9.2 ± 0.2	61.0 ± 0.5	19.4 ± 0.2	10.4 ± 0.3	-	-	-	-	-
Mn^2+^/Zn^2+^/Fe^3+^/Mg-Al-LDH	8.4 ± 0.3	45.5 ± 0.5	12.7 ± 0.1	10.2 ± 0.3	-	9.4 ± 0.5	3.5 ± 0.3	7.2 ± 0.4	3.2 ± 0.3
P-loaded Mn^2+^/Zn^2+^/Fe^3+^/Mg-Al-LDH	8.5 ± 0.4	59.1 ± 0.4	11.4 ± 0.2	10.4 ± 0.3	3.2 ± 0.3	0.7 ± 0.2	1.7 ± 0.2	3.0 ± 0.3	2.1 ± 0.3

**Table 2:** Comparison of phosphate adsorption capacities of some LDHs adsorbents.

Adsorbent	Description	Nomenclature	$q_{m}$(mg∙g^−1^)	Condition	Ref.
Parent Mg-Al-LDH	Synthetized Mg-Al-LDH	Mg-Al-LDH	65.3	20 °C, pH 7.5	This study
Mn^2+^/Zn^2+^/Fe^3+^/Mg (oxy)hydroxide nanoparticles supported onto parent Mg-Al-LDH	Metals (oxy)hydroxide nanoparticles supported onto parent Mg-Al-LDH	Mn^2+^/Zn^2+^/Fe^3+^/Mg-Al-LDH	82.3		
FeMgMn-LDH	FeMgMn-LDH synthesized in a single co-precipitation step.	FeMg_2_Mn-LDH	34.3	25 °C, pH 6.5	[26]
MgAl-NO_3_-LDH	Synthetized Mg-Al-LDH in a single co-precipitation step.	MgAl-NO_3_-LDH	64.1	25 °C, pH 6	[44]
MgAl-Cl-LDH	Synthetized Mg-Al-LDH in a single co-precipitation step.	Mg-Al LDH	69.8	25 °C, pH 5	[17]
Fe-Mg-LDH	Purchased Fe-HT3.0 and Fe-HT5.0	Fe-Mg-LDH	58.3	-	[23]
Zn-Al-LDH	Zn-Al-LDH synthesized in a single co-precipitation step.	Zn-Al-70-LDH	20.7	25 °C, pH 6.8	[22]
Natural clays	Natural form of clays	C_1_	21.4	20 °C, pH 7.0	[2]
		C_2_	20.9		
	Iron-doped clays	C_1_-Fe	38.0		
		C_2_-Fe	37.6		
Hydrothermally synthesized zeolites	Iron-doped zeolites	LTA-Fe	18.5	20 °C, pH 7.0	[5]
		FAU-X-Fe	17.5		
Natural zeolites	Al/Fe/Mn doped clinoptilolite	ZN	0.6	20 °C, pH 7.0	[11]
		Z-Al	7.0		
		Z-Fe	3.4		[45]
		Z-Mn	5.6		[46]

**Table 3:** Isotherm parameters of the phosphate adsorption onto Mn^2+^/Zn^2+^/Fe^3+^/Mg-Al-LDH composite.

Temperature (K)	Langmuir			Freundlich		
	$q_{m}$ (mg·g^−1^)	$k_{L}$ (L·mg^−1^)	R^2^	n	$k_{F}$ (mg·g^−1^)	R^2^
293.15	82.34	0.03	0.99	3.10	8.65	0.73
298.15	86.45	0.04	0.99	3.23	9.13	0.75
303.15	87.98	0.05	0.99	3.45	10.1	0.77

**Table 4:** Thermodynamic parameters of the phosphate adsorption onto Mn^2+^/Zn^2+^/Fe^3+^/Mg-Al-LDH composite.

Temperature (K)	$\ln\;k_{c}$	R^2^	∆G	∆S	∆H
			(kJ·mol^−1^)	(kJ·mol^−1^·K^−1^)	(kJ·mol^−1^)
293.15	11.97	0.99	−29.18	0.23	37.77
298.15	12.26		−30.39		
303.15	12.48		−31.46		

**Table 5:** Kinetic parameters of the phosphate adsorption onto Mn^2+^/Zn^2+^/Fe^3+^/Mg-Al-LDH.

Model	Kinetic Parameters	Phosphate
Pseudo-first order	$q_{e}$ (mg·g^−1^)	10.5
	$k_{1}$ (h^−1^)	0.2
	R^2^	0.76
Pseudo-second order	$q_{e}$ (mg·g^−1^)	32.7
	$k_{2}$ (g·mg^−1^·h^−1^)	0.19
	R^2^	0.99
Intraparticle diffusion	$k_{t1}$ (mg·g^−1^·h^−1/2^)	29.7
	R^2^	0.77
	$k_{t2}$ (mg·g^−1^·h^−1/2^)	1.9
	R^2^	0.90
	$k_{t3}$ (mg·g^−1^·h^−1/2^)	1.9
	R^2^	0.95
HPDF Film diffusion	$D_{f}$ (m^2^·^s^^−1^)	5.6 × 10^−15^
	R^2^	0.93
HPDM Particle diffusion	$D_{p}$ (m^2^·s^−1^)	2.6 × 10^−14^
	R^2^	0.90

**Table 6:** Phosphate adsorption capacity onto Mn^2+^/Zn^2+^/Fe^3+^/Mg-Al-LDH composite in presence of competing ions.

Anion	$q_{e}$ (mg·g^−1^)	q^mix^/q^0^
PO_4_^3^^−^	12.4 ± 0.3	-
PO_4_^3^^−^ + NO_3_^−^	17.6 ± 0.4	1.4
PO_4_^3^^−^+ SO_4_^2^^−^	11.7 ± 0.4	0.9
PO_4_^3^^−^ + Cl^−^	12.3 ± 0.5	1.0
PO_4_^3^^−^ + HCO_3_^−^	13.7 ± 0.4	1.1
PO_4_^3^^−^ + All anions	10.4 ± 0.6	0.8

**Table 7:** Chemical forms of phosphate extracted from loaded Mn^2+^/Zn^2+^/Fe^3+^/Mg-Al-LDH composite.

$q_{e}$	LB-P		(Fe + Zn + Mn)-P		(Mg)-P		R-P	
(mg·g^−1^)	(mg·g^−1^)	%	(mg·g^−1^)	%	(mg·g^−1^)	%	(mg·g^−1^)	%
18.0	2.5	14 ± 1	9.2	51 ± 3	5.8	32 ± 2	0.5	3 ± 1

## Data Availability

Not applicable.
